# The normal impact stiffness of a debris-flow flexible barrier

**DOI:** 10.1038/s41598-023-30664-2

**Published:** 2023-03-09

**Authors:** Miao Huo, Jia-wen Zhou, Jiangtao Zhao, Hong-wei Zhou, Jidong Li, Xing Liu

**Affiliations:** 1 College of Water Conservancy and Hydropower Engineering, Sichuan Agricultural University, Ya’an City, 625014 China; 2 State Key Laboratory of Hydraulics and Mountain River Engineering, Sichuan University, Chengdu, 610065 China

**Keywords:** Civil engineering, Natural hazards

## Abstract

This paper proposes a normal oriented impact stiffness of a three-supporting cable flexible barrier under a small pretension stress to estimate the structural load behaviour, and employs two categories of small-scale debris flows (coarse and fine) to explore the stiffness evolution through physical model experiments with high-speed photography and load sensing. Results suggest that the particle-structure contact is essential to the normal load effect. Coarse debris flow performs more frequent particle-structure contact and exerts evident momentum flux, while fine debris flows with few physical collisions impart much smaller one. The middle-sited cable that receives only tensile force from vertical equivalent cable-net joint system exhibits indirect load behaviour. The bottom-sited cable shows high load feedback due to the sum of direct contact of debris flow and tensile forces. The relationship between impact loads and maximum cable deflections can be explained by power functions according to quasi-static theory. The impact stiffness is not just affected by the particle-structure contact but by the flow inertia and particle collision effect. Savage number *N*_*sav*_ and Bagnold number* N*_*bag*_ manage to depict the dynamical effects on the normal stiffness *D*_*i*_. Experiments indicate that *N*_*sav*_ has positive linear correlation with the nondimensionalization of *D*_*i*_, whilst *N*_*bag*_ has positive power correlation with the nondimensionalization of *D*_*i*_. This idea is an alternative scope for the study on flow-structure interaction and may contribute to the parameter identification in numerical simulation of the debris flow-structure interaction and the optimization of the design standardization.

## Introduction

The frequency of landslides or debris flows is high in the mountainous areas of Southwest China due to steep passages, abundant rainfalls and solid-fragment sources^1^. As affected by the surge of extreme rainstorm events worldwide in recent years, some low frequency-landslides areas have turned into high ones, and the scale of hazards increases, which is difficult to accurately estimate and is causing considerable threats to residents and infrastructures nearby, as well as difficulties in the design of prevention and control measures.

Flexible barrier is a valid measure for small-scale debris flow retaining. Its light and open overall structure makes the impact of natural environment small, and makes the construction fast and economical. Therefore, it meets the requirements of multi-point treatment of debris-flow gullies in southwest mountainous areas of China, and has a promising prospect^2–5^. However, structural response under debris-flow impact is still progressing due to the complex geometric nonlinearity of the flexible barrier^6–8^. Currently, the stiffness of flexible barrier against debris flow is not clear, and the conventional design of the structure in China is mainly on the basis of a conservative energy dissipation model. In practice, the wire net component of the structure may be breached prior to the failures of supporting cables or anchorages^9–11^, indicating a gap between structural design theory and actual engineering function. It is theoretically recognized that the internal shear stress and bending moment of the cable-net structure can be ignored due to the remarkable tension feature. The debris-flow impact is dispersed and transmitted by the tension of the cable-net structure so researches on the structural stiffness of flexible barrier focus on the tensile force and deformation. Ashwood^12^ uses axial stiffness of a cable as a key parameter that is based on linear relationship between load and deflection to quantify the flow-structure interaction. Large boulders or coarse particles are inclined to accumulate in the head of a debris flow, and the load distribution of the structure under high-speed thrust is transient. So the stiffness in the direction of the surge should serve as another factor of mechanical stability. The relevant knowledge of structural load distribution is mostly solved by quasi-static method that decomposes the impact of debris flow into dynamic load and static earth pressure load, but the cross section of the structure is defined as a cantilever beam, ignoring the impact of thrust-direction deformation^13^. Normal stiffness that is perpendicular to the stretch face of a flexible barrier tends to demonstrate the shear resistance of the cable-net system during instant impacts. Ng et al.^14,15^ have already put load–displacement behaviour of the supporting cable into normal stiffness analysis. Song et al.^16^ studied the maximum normal stiffness of supporting cable under distributed and concentrated load by adding the Froude number of debris flow *F*_*r*_ and found larger maximum normal stiffness under distributed load. A follow-up study discussed the flow regime and the compressibility of dry granular flows encountering a flexible barrier in depth, indicating that the deflection of flexible barrier and the state of granular material both contribute to the variation of impact load^17^. In addition, the arresting cables on an aircraft carrier are identified as a structure with tensile and bending stiffness, and the developing mode of tangential stress that is normal to the cable during the arresting process emphasizes the necessity of tangential stress analysis of cable structure under high-speed impact^18^.

Debris-flow impact on an anti-structure can be reviewed in many literatures^19–22^, but the structural deformations differ from each other with respect to varied textures. Since structural stiffness is a link between load and deformation, form-finding of flexible barrier is the primary process for identifying stiffness. Methods such as force-density, dynamic relaxation are performed to assist with form-finding^23,24^, whereas effects of time-variant normal displacement of nodes and pretension reservation are complex, which hinders the development. Albrecht and Volkwein^25^ studied the dynamic response of a flexible barrier with rhombic net subjected to concentrated impact load, and defined the deformation state of the structure under initial and impact process by using the stiffness of the net component, then the failure characteristics of the structure under concentrated load are described. The results show that the partial area that endures direct contact-impact is the commencement of structural failure. Jiang et al.^26^ figure that the direct particle-impact on the structure leads to non-regular parabola deformation, while the non-contact part receives parabolic displacement. Song et al.^27^ correlated the normal impact force with the debris flow surge assessment based on the load model proposed by Song et al.^28^ and tensile force detection, providing a good approach to presenting flow-barrier interaction. And full scale tests have taken the development of normal impact force and displacement as a factor to display load path before the failure state^29^.

**Figure 1:**
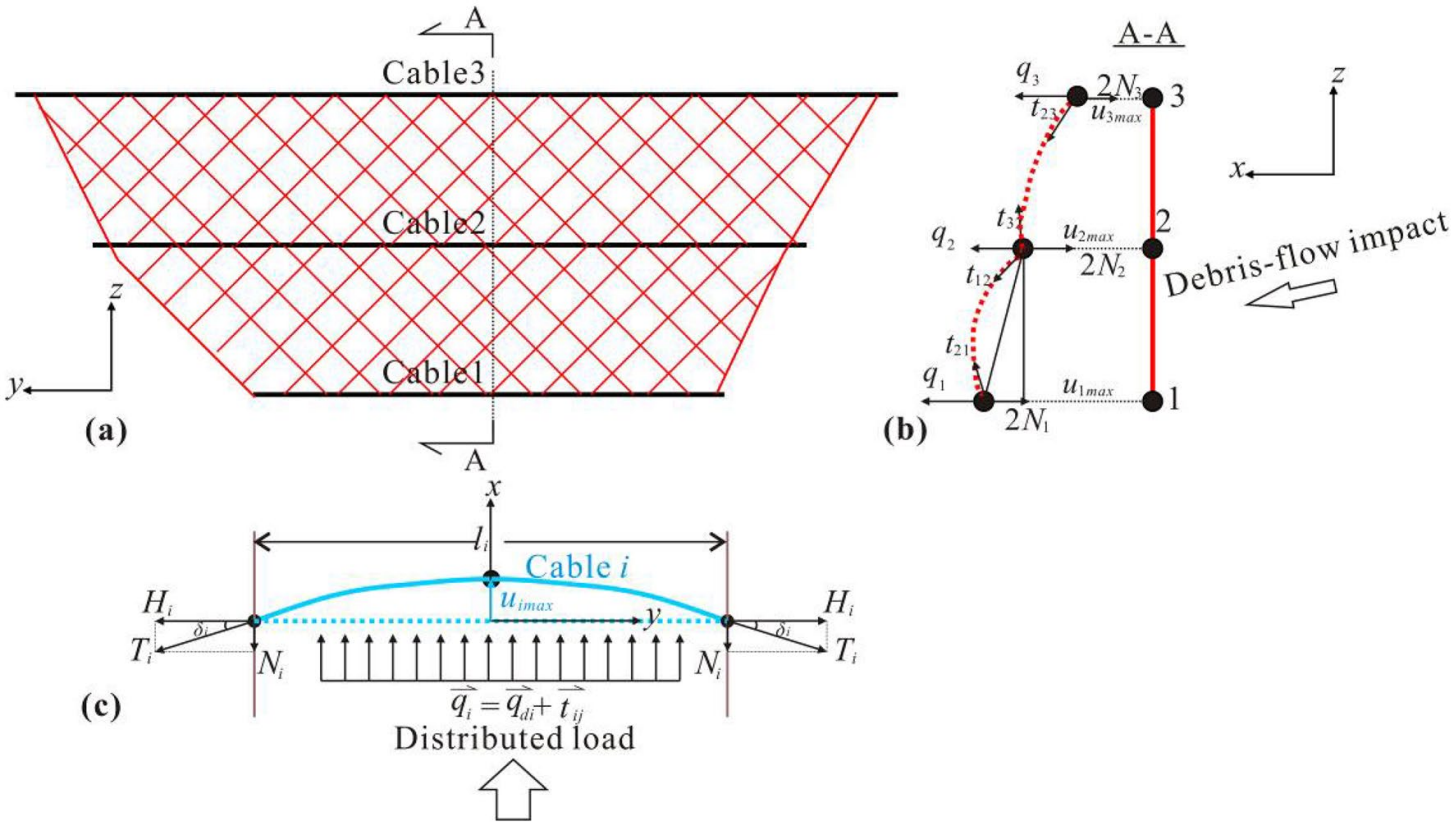
Systematic diagram of the impact model: (**a**) frontal view of a flexible barrier, (**b**) cross-section deformation of the flexible barrier subjected to debris-flow impact and (**c**) the maximum deflection of a supporting cable under impact load *u*_*imax*_.

According to tension structure mechanism^30^, tensile force model can be envisioned as one with national shear stress and bending moment to solve the load-deformation problem, which indicates the rationality of normal impact model. This study proposes a preliminary theory about cable stiffness that is quantified by debris-flow distributed load and maximum deflection along the cable. And time-varying and nonlinear behaviour of the distributed load on the structure based on compatibility criterion is applied to verify this stiffness model. The theory stems from quasi-static impact mode between a debris flow and a flexible barrier and follows the idea proposed by Brighenti et al.^31^. The evaluation of the stiffness is indicated by the maximum normal deflection of a supporting cable and its perpendicular distributed load, meanwhile, the distributed load should cover the one that is transmitted by vertical equivalent cable-net joint system i. e. the pulling force exerted from other cables and the net. In addition, some simplifications are conducted to make the model handy: (i) the debris-flow impact vector is normal to the initial cable direction, (ii) the whole structure is elastic and (iii) the deflection of the cable is parabolic with the width. Thus, the elaboration of the stiffness model is displayed in Fig. 1.

As shown in Fig. 1c, by taking the quasi-static notion and the coordination of single cable and deformation into consideration, the distributed load on the cable *i* is expressed as follow:

1 $$q_{i} l_{i} = 2N_{i} = 2H_{i} \tan \delta _{i} = 2T_{i} \sin \delta _{i}$$

where* q*_*i*_ denotes distributed load perpendicular to the supporting cable *i*. *N*_*i*_ and *H*_*i*_ are the tangential and horizontal components of supporting reaction force of the anchorage, respectively. *T*_*i*_ is the tensile force that is acting on the anchorage. And *δ*_*i*_ is extremity-deflection angle of cable *i*.

Provided that the cable *i* is in the fully tensioned state, *q*_*i*_ meets the following correlation

2 $$q_{i} = \frac{{64E_{i} A_{i} u_{i\max }^{3} }}{{3l_{i}^{4} }}$$

*Ei* and *Ai* are elastic modulus and cross section of the cable *i*, separately. *ui*max is the maximum deflection of the cable *i*. Aiming to interpret the debris-flow impact, *qi* need to be decomposed into the debris-flow impact load *qdi* and the sum of impact direction components of tensile loads transmitted from the cable-net joint system *tjh*:

3 $$q_{i} = q_{di} { + }t_{jh}$$

Ultimately, the debris-flow impact stiffness of a cable can be expressed as the distributed load imparted from a debris flow and the maximum cable deflection:

4 $$D_{i} = \frac{{q_{di} }}{{u_{i\max } }}$$

*D*_*i*_ (N/m^2^) contains tensile elastic modulus *E*_*i*_*A*_*i*_ and reflects the resistance of the structure to the frontal impact of a debris flow theoretically. However, *t*_*jh*_ can hardly get an analytic solution due to the nonlinear behaviour of the vertical cable-net cross section. So this paper provides a specific experiment to eliminate the effect of *t*_*jh*_. The load exerted by the debris flow *q*_*di*_ is deduced by the profile deformation compatibility of the vertical cable-net system, which is already presented in^26^.

Above all, the stretch face-normal load and deflection both play direct roles in describing the debris flow impact. The presented study aims to explore a normalised impact stiffness of the supporting cable without energy dissipators based on the aforementioned theory. Physical structure model tests are conducted to analyze the interaction between small-scale debris flows and flexible barrier with large opening and exclude the tensile load transmission.

## Results

**Figure 2:**
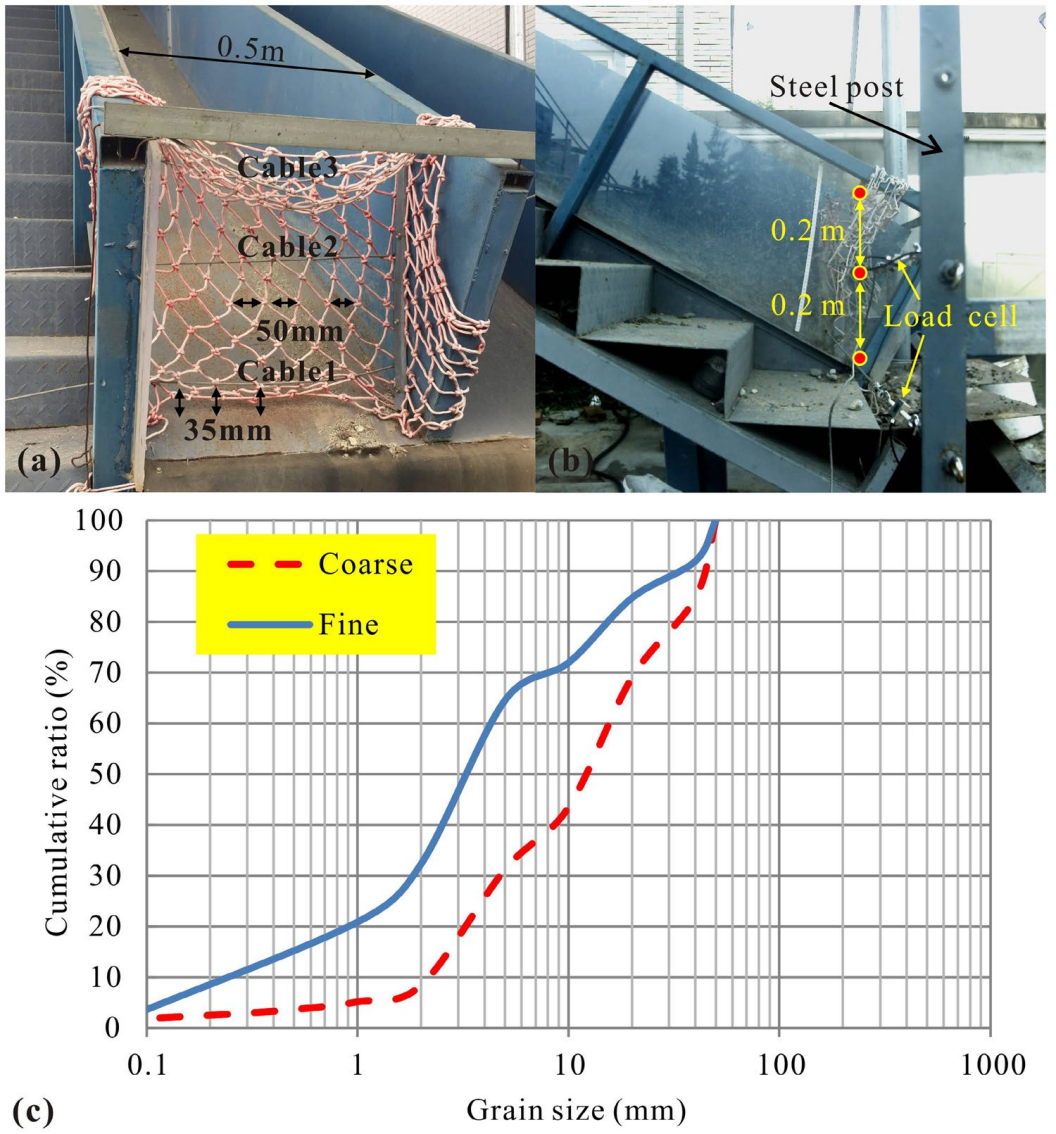
Experimental model: (**a**) the frontal and (**b**) the profile shooting of flume facility with the F. B. and (**c**) grain size distribution of the sediment.

Through small-scale experiments with specific design, structural behaviours derived from impact forces exerted by two categories of model debris-flow i.e. coarse and fine, have managed to verify the key function of the stiffness. The flexible barrier model (abbreviated as F. B. model) is parallel to the gravity, thus the load effect is more distinct than where the F. B. is perpendicular to the flume bottom line^40^. The F.B. is 0.4 m high and 0.5 m wide with three equally adjacent supporting cable models with circular section (the diameter of the section is 4 mm) namely top, intermediate and bottom cable, and the cables are interlaced with the net. Texture of the net in the F. B. is nylon, winding into a rhombic-mesh rope-wire system. The mesh size is open enough (equal to the maximum grain size of the debris-flow material) in case of sediment reaching the intermediate cable’s height (Fig. 2a). The extremity of one cable is bolted with a load cell (100 g of mass, 100 times per second of sampling rate and 0.1 N of resolution) and are anchored to a vertical steel post that is fixed on the right side of the flume (Fig. 2b). Intermediate and bottom cables are both extended out of the flume (length of 1.1 m versus width of 0.5 m) in order to provide more space for energy dissipation^27^. Furthermore, the pretension force of the intermediate and the bottom cable is set to an identical value (approximately 0.01 N and the pretension stress is 796 Pa), hence the initial stiff state of each cable is equal for better comparison during impact processes.

The whole impact process is no more than 5 s, and the ultimate sediment behind the F. B. is a little lower than the height of the intermediate cable. This indicates that the bottom cable bears the direct contact of debris flows while the top and intermediate cables are only subjected to the tensile force of vertical cable-net joint system. The load behaviour of the top cable is not concerned due to no load cell being planted upon it. And neither is the test group FL owing to the failure of tension force detection. More details on experimental procedure can be seen in “Experimental procedure” section.

### Flow and load behaviour

**Figure 3:**
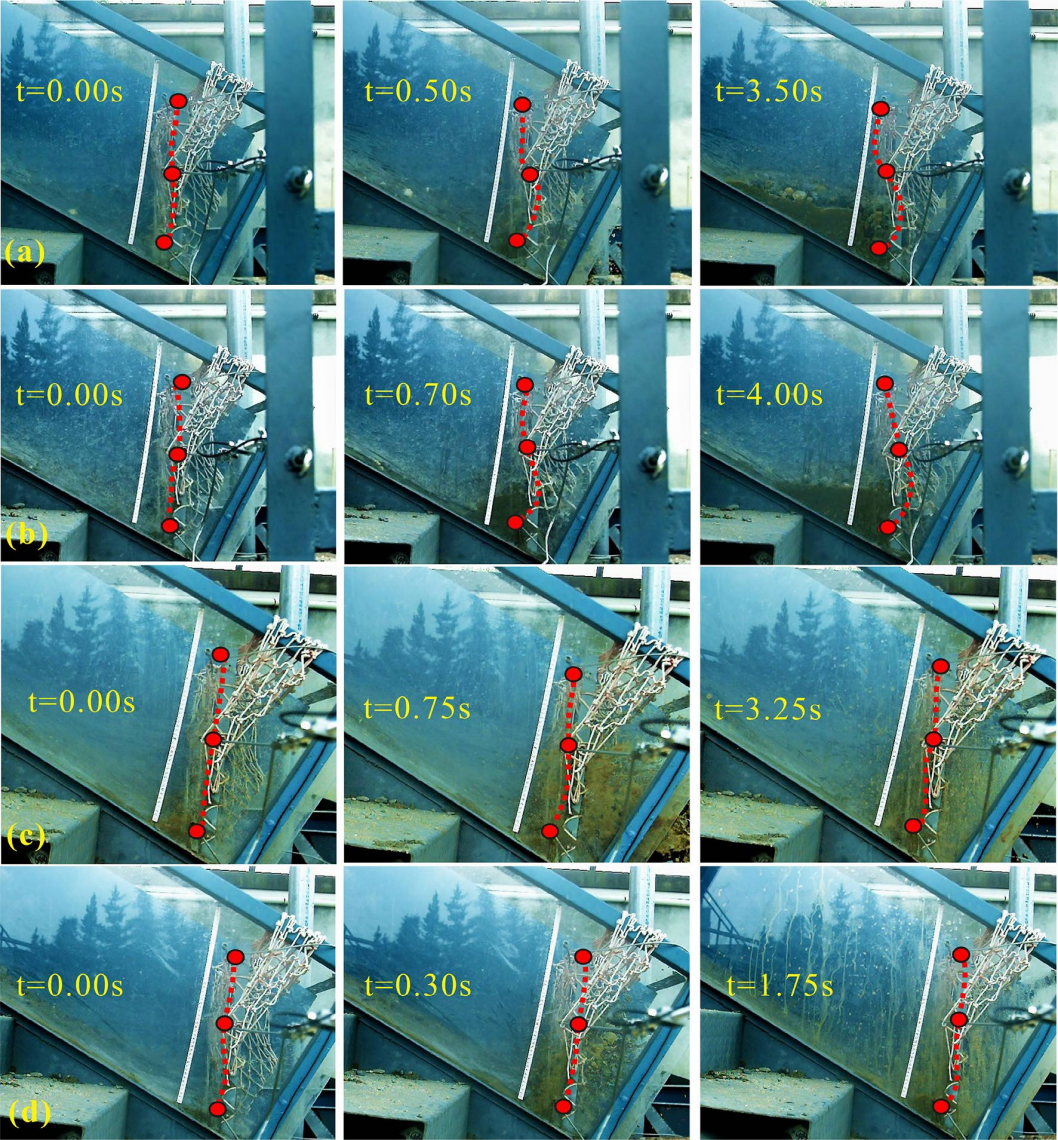
Deflection of the F. B. subjected to time-depended impacts: (**a**) CL, (**b**) CS, (**c**) FL and (**d**) FS.

**Figure 4:**
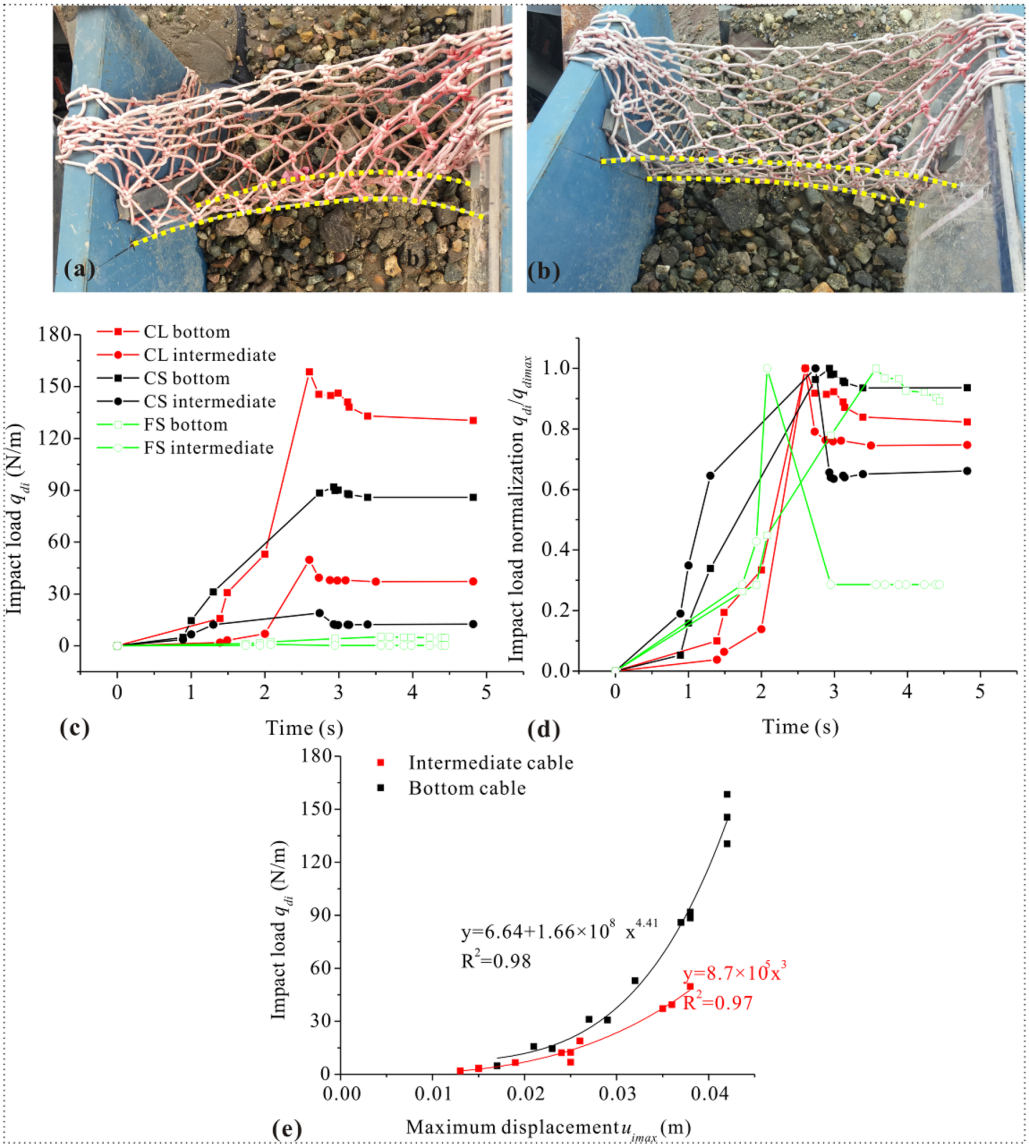
(**a**) The cable deflection pattern of CL (**b**) and CS, (**c**) distributed impact load *q*_*di*_ versus time (**d**) normalised distributed impact load namely the impact load by the maximum load $\frac{{q_{di} }}{{q_{dimax} }}$ versus time and (**e**) distributed debris-flow impact load *q*_*di*_ versus maximum deflection *u*_*imax*_.

Multiple parameters of the model debris flow are documented during the impact process, covering the average approaching velocity *U*, flow thickness *h*. There is no significant difference between kinematic parameters of coarse and fine debris flows, and even the values of *U* and *h* in the fine debris flow category surpass the ones in the coarse category (Table 1). On the other hand, the sediment behind the barrier poses a run-up mode and never be able to climb over the height of the intermediate cable in each test (Fig. 3). The ultimate outflow rate in weight is 0.21, 0.13, 0.61 and 0.56 in test CL, CS, FL and FS, respectively, which proves that the perviousness of the F.B. model is not only related to the maximum grain size but also the majority of the particle size distribution. The Froude number *Fr* of approaching flows is back-calculated according to *U* and *h* at certain moments, and it varies with different setups and impact durations (Table 1). Generally, values of *Fr* indicate that the flows are dominated by gravity and inertia forces, and the range of values follow multiple natural debris flows^41,35^. Figure 3 also shows deflection profiles during typical impact processes, which are divided into initial, interacting and static stage. It is found that the cables are basically deflected along the normal-impact direction, and the maximum deflection increases from top to bottom, and the retraction is not obvious afterwards. The coarse category poses greater deflections compared with the fine category. The fine category generates almost slurry impact despite minority of coarse particles (10% of which fall in the range of 35–50 mm) ahead. The deflection pattern along the cable is parabolic with the width (Fig. 4a,b), corresponding to the abovementioned hypothesis.

**Table 1:** Kinematic parameters of the experimental debris flows and the impact stiffness *D*_*i*_ of the cable.

Test NO	Time (s)	*D*_50_ (mm)	Velocity *U* (m/s)	Flow thickness *h* (m)	*Fr*	*N*_*sav*_	*N*_*bag*_	Intermediate *D*_2_ (N/m^2^)	Bottom *D*_1_ (N/m^2^)
CL	1.39	13	1.58	0.021	3.48	9.42	40.94	147	753
CL	1.49	13	1.61	0.018	3.83	15.57	48.71	212	1061
CL	2.00	13	1.70	0.020	3.82	12.43	46.01	274	1657
CL	2.60	13	3.63	0.023	7.64	37.44	85.64	1310	3774
CS	0.89	13	1.59	0.026	3.14	4.97	33.18	237	286
CS	1.00	13	1.83	0.021	4.03	12.50	47.28	349	632
CS	1.30	13	1.44	0.019	3.34	10.45	41.12	509	1153
CS	2.93	13	3.14	0.023	6.61	28.01	74.08	757	3401
FS	1.74	3.5	2.59	0.029	4.86	0.69	3.51	18	170
FS	1.93	3.5	2.31	0.032	4.12	0.41	2.84	27	123
FS	2.08	3.5	2.95	0.033	5.18	0.61	3.52	52	193
FS	2.95	3.5	2.19	0.024	4.51	0.87	3.59	12	174

The values in the same group vary because relative data is logged in different duration.

The impact load and stiffness of the fine category drop violently. As shown in Fig. 4c, the *q*_*di*_ reaches its peak value during the early impact process, and then drops to constant value and maintains static state. The distributed impact load in FS group is much smaller than that in coarse category and the normalised load also drops more violently in FS group (Fig. 4d), indicating that the less contact frequency with the cable-net network and the larger permeability make the structural response insensitive in FS group. The impact of coarse particles on the cable structure shows larger load feedback, which emphasizes the importance of the contact between particles and the structure. This trend varies similarly with descriptions of barriers in some literatures^21,27,35^. On the other hand, particle-structure contact is secured in the coarse category, so debris flow with longer moving distance tends to have more powerful impact force. What’s more, *q*_*di*_ on the bottom cable always remains the greater compared with that of the intermediate one, thus it implies that the cable stiffness would be different under different degrees of impact.

Since the intermediate cable is not in direct contact with debris flow but is only subjected to tensile force, the normal impact load should be a power function of the maximum deflection according to Eq. (2), that is:

5 $$q_{i} = a \cdot u_{i\max }^{b}$$

where coefficient *a* and *b* feature the development. If the cable is completely tighten, coefficient *a* and *b* can be calibrated by bringing the standard value of elastic modulus (*E* = 2.06 × 10^11^ Pa) in Eq. (2). Regression through experimental data on the intermediate cable is also the power function like Eq. (5), the correlation coefficient of which is 0.97 after the coefficient *b* is set to 3 (Fig. 4e). But back-analysis of the coefficient *a* reflects much lower scale (*a* = 8.7 × 10^5^ Pa·m^−2^).

As the complexity of the theoretic expression, we apply regression to describe the load–deflection behaviour of the bottom cable based on load cell data. And the most appropriate is another form of power function:

6 $$q_{i} = c + d \cdot u_{i\max }^{e}$$

where the coefficient *c* = 6.64, *d* = 1.66 × 10^8^ and *e* = 4.41 with correlation coefficient of 0.98 (Fig. 4e). The coefficient *c* denotes the normal component of the load from vertical cable-net joint system. Above mentioned results demonstrate the key role of the solid contact in the impact load behaviour and the validity of the framework that is co-expressed by Eqs. (2) and (3). And coefficient *d* and *e* are not equal to theoretical values due to coarse and fine flow-motion impacts.

### Normal stiffness development

Given that the coarse and fine categories of debris flow have distinct impacts on the cable structure, the normal impact stiffness is investigated with some indexes that feature debris-flow motions. Details on the dimensional analysis are introduced in “Scale principle and dimensional analysis” section.

**Figure 5:**
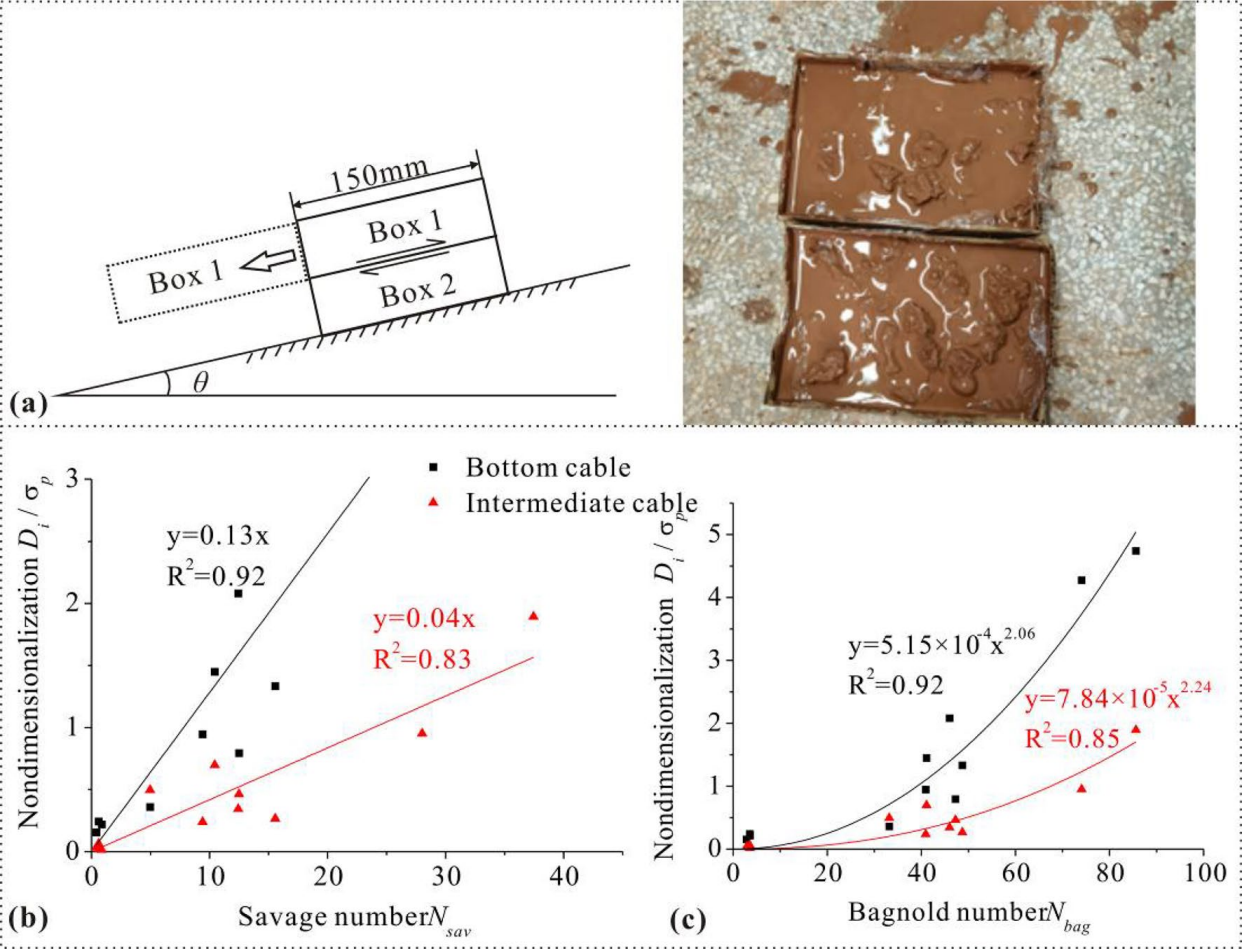
(**a**) two boxes full of debris-flow material for the internal friction angle testing, (**b**) the ratio of normal stiffness *D*_*i*_ to pretension stress *σ*_*p*_ versus Savage number *N*_*sav*_ and (**c**) versus Bagnold number *N*_*bag*_.

First, the savage number of debris flow *N*_*sav*_ is introduced (Table 1). It is a quantify the extent of solid particle inertia force and friction collision force on motion within a debris flow^33,34^. The computational expression of *N*_*sav*_ is as follow:

7 $$N_{sav} = \frac{{\rho_{s} \delta^{2} \gamma_{k}^{2} }}{{\left( {\rho_{s} - \rho_{f} } \right)gh_{{}} \tan \varphi }}$$

where ρs and ρf are the density of solid and fluid in the debris flow, respectively, and δ denotes particular grain size that dominants major movement of particles, which can be substituted by average grain size D50. γk is the shear rate along the flow thickness, estimated by $\frac{U}{h}$^33,34^. φ is the internal friction angle of the model debris flow material. φ is preliminarily tested through a pair of two boxes (without cap) filled with model debris flow material, and the two boxes are stacked on top of each other and placed in a small chute with adjustable inclination (Fig. 5a). After steepen the inclination, the box on the top would begin to slide and separate from the one underneath. Associated with the timing t, the sliding inclination of the chute θ and the longitudinal length of the box S, the φ can be derived from the Eq. (8) below:

8 $$2S = \left( {g\sin \theta - \mu g\cos \theta } \right)t^{2}$$

Here by simple calculation, *φ*≈39°and *φ*≈36°in coarse category and fine category. *φ* remains static during impact. Furthermore, the pretension stress is also a factor that affects the initial stiffness, thus we nondimensionalize the *D*_*i*_ as the ratio of *D*_*i*_ to the pretension stress *σ*_*p*_ namely $\frac{{D_{i} }}{{\sigma _{p} }}$, for possibly general utilization. The *σ*_*p*_ is envisioned as a characteristic stress. Fitting curve indicates $\frac{{D_{i} }}{{\sigma _{p} }}$ grows linearly with *N*_*sav*_ (fitting curve expression is $\frac{{D_{1} }}{{\sigma_{p} }}$ = 0.13*N*_*sav*_, with *R*^2^ = 0.92 and $\frac{{D_{2} }}{{\sigma_{p} }}$ = 0.04*N*_*sav*_, with *R*^2^ = 0.83) and the normal impact stiffness of intermediate cable receiving indirect load is lower than that of bottom cable when incoming flow motion inertia is equal (Fig. 5b).

The Bagnold number *N*_*bag*_ is perceived as a valid factor to depict internal particle collision of a debris flow. Here we harness *N*_*bag*_ for seeking another feasibility of particle-structure contact to describe the stiffness development (Table 1). The calculation of *N*_*bag*_ is elaborated in literature^33^ and nondimensionalization of *D*_*i*_ is also employed. It is found that $\frac{{D_{1} }}{{\sigma_{p} }}$ and $\frac{{D_{2} }}{{\sigma_{p} }}$ both increase in a power growth with *N*_*bag*_ in a certain range (Fig. 5c). Compared with the effect of *N*_*sav*_, the increasing rate of $\frac{{D_{i} }}{{\sigma_{p} }}$ ascends with *N*_*bag*_. The power fitting expressions are $\frac{{D_{1} }}{{\sigma_{p} }}$ = 5.15 × 10^–4^ (*N*_*sav*_)^2.06^ with *R*^2^ = 0.92 and $\frac{{D_{2} }}{{\sigma_{p} }}$ = 7.84 × 10^–5^ (*N*_*sav*_)^2.24^ with *R*^2^ = 0.85. Similarly, the normal impact stiffness of intermediate cable receiving indirect load is lower than that of bottom cable when debris flow internal particle collision effect is the same.

## Discussion

A novel framework for assessing normal debris-flow impact stiffness of a flexible barrier is introduced in this paper through physical model experiments. The normal impact stiffness is an alternative estimation of the distributed impact load of a debris flow, which can be perceived as the shear stiffness mentioned in literature^35^, but with different dimension. This cable stiffness is mainly used to profile the impact load from debris flow and pulling force transmitted by the vertical equivalent cable-net joint system which is very hard to estimate in practice. The effect of the stiffness on the load distribution needs to be further explored by numerical simulation and add energy dissipation component.

Debris flows with different grain size distributions can pose different loads on the F. B. model. The coarse particles tend to accumulate in the head of the coarse category debris flow and impart larger force than that of fine debris flows more particle-structure contact. Normal impact load is estimated on quasi-static theory and tension data detected by load cells. And load transmission shows that cables in different heights endure very different forces. Great load acts on the bottom cable for the sum of direct impact of debris flow and drugging tensile force from equivalent vertical cables i. e. indirect force. As for the intermediate one that receives only indirect force, the load feedback is surely low. Literature 28 reported a four-cable flexible barrier model receiving highest impact force in lower intermediate cable instead of the bottom cable in this study. But the bottom cable here is 35 mm far to the baseplate, which also indicates the idea that the lower part of the F.B. model is subjected to greater impact force. Experimental relationship between impact load and maximum deflection is meets the power-function pattern that explained by the theoretical deduction from Eq. (2), confirming the applicability of the quasi-static theory. This pattern is roughly consistent with the force–displacement mode of chain-link net barrier explained by Ng et al.^14,15^ and Escallon et al.^29^. However, all cables within the F. B. are not flawlessly tighten, inducing the calculated value of elastic modulus through Eq. (5) to be far less than the standard value of elastic modulus (*E* = 2.06 × 10^11^ Pa). This trend enables the structural elastic modulus in the loose state to be a particular index to control pretension force in practice.

The velocity and thickness of the fine debris flow differs less from those of the coarse one under the same moving distance condition, indicating almost the same incoming flow state. Nevertheless, normal load and stiffness under fine debris-flow impact are extremely low, which infers particle contact with the structure could be crucial to the stiff performance. Therefore, we introduce the motion inertia and the particle collision effect represented by *N*_*sav*_ and *N*_*bag*_, respectively, to explore the development of the normal stiffness *D*_*i*_. And the average particle size *D*_50_ of coarse and fine debris flows within the two factors is also proved to be valid in assessing the impact load. The positive relations between these two factors and *D*_*i*_ eventually verify the hypothesis that flow motion inertia and particle-contact with the structure both contribute to the increasing of *D*_*i*_ within a certain range. There is a deduction that these positive connections will be invalid after *D*_*i*_ exceeding a certain value which is related to the critical state of failure^29^. Noting that $\frac{{D_{i} }}{{\sigma_{p} }}$, *N*_*sav*_ and *N*_*bag*_ are all dimensionless, so scale effects might be ignored when compared with practice engineering. But experimental error and static estimation of parameters may impact the accuracies of the two relation functions. Intensive study should be carried out to obtain perfect parameter inversion within the fitting curves. To sum up, this integrated insight of flow motion and particle-structure contact is essential to the load mechanism of a debris-flow flexible barrier, which shares the same opinion of solid fraction impact on a barrier^35^. The intermediate cable of a flexible barrier serves as a cushion when imposed by indirect tension load, indicating distributing load effect of vertical net wire^36–38^. On the other hand, direct load from particle-slurry impact along with tension load from vertical wires makes the bottom cable stiffer. Therefore, the reinforcement of bottom cable or lower part is recommended in practice. The experimental analysis is an active attempt to figure out debris-flow impact evolution on flexible barrier for ultimately securing the critical state to be near ductile failure instead of brittle failure. Experiments lack of further discussion on the effects of cable braking system, structure-failure modes and varied pretension stresses, so subsequent study will cover these aspects.

The abovementioned estimation and results may provide a theoretical reference for the load calculation and a rational verification of related stiffness parameter in a numerical simulation plan.

## Methods

### Experimental procedure

The experiment procedure is carried out in the same flume model facility introduced by Jiang et al.^26^. The inclination of flume is 30° and the F. B. model is erected at the exit of the flume. 100 kg of water-saturated sediment with two kinds of grain size distribution is released from two different moving distances secured by two uplifted gates (3.2 m abbreviated as S and 5.0 m abbreviated as L) to hit the F. B. model. The mean diameters of two debris flow models are 13 mm and 3.5 mm, respectively, representing coarse and fine categories (Fig. 2c). The basal gap (between bottom cable and baseplate) is set to 35 mm, which is equal to the maximum diameter of 90% of the grains in coarse groups^39^. The procedure provides four experimental loading groups in total (Table 2). Besides, a high-speed camera (200 frames per second) is employed laterally and a digital camera is planted topside to monitor the whole impact and structural deformation process. Flow and structure profiles are documented, and then kinematic parameters such as flow velocity, thickness, *Fr*, *N*_*bag*_ and *N*_*sav*_ are back-calculated from camera shooting interval and image size calibration. Values of the approaching velocity and thickness are counted inside a region of 0.5 m by 0.5 m near the F.B. model.

**Table 2:** Experimental settings.

Status of material	Total mass (kg)	Bulk density (kg/m^3^)	Maximum diameter *D*_*max*_ (mm)	Mean diameter* D*_50_(mm)	Solid volume fraction	Moving distance (m)	Test NO
Coarse	100	1680	50	13	0.5	5.0	CL
						3.2	CS
Fine		1810	50	3.5	0.5	3.2	FS
						5.0	FL

Before each test, debris material is stirred with water repeatedly in a storage section which consists of the upstream side of the flume and an uplifted gate, and then it is released by a dam-breach fashion. When debris flows impact the F. B. model, the load cells and the high-speed camera are triggered simultaneously to ensure the tensile loads and structural deflections to be synchronized. Eventually, the impact that denotes the normal distributed load acting on the initial length (within the span) *q*_*di*_ is obtained by Eqs. (1) and (3).

### Scale principle and dimensional analysis

The Froude number *Fr* dominates the dynamical similarity between the small-scale tests and field engineering. Besides, no centrifuge is employed here, thus density and gravity are also controlled (Table 3). Debris flow and structure of the F.B. model are highly nonlinear and time-varying. Physical scenario of the flow-structure interaction is very complicated and flow kinematic parameters cannot be directly introduced. Whilst effect of *Fr* on the barrier-impact load has been well established in many literatures^27,40,35,43^, other two dimensionless parameters i.e. *N*_*sav*_ and *N*_*bag*_ that describe the flow momentum transport should also be examined. In addition, some structural parameters based on the quasi-static theory are input into dimensional analysis:

9 $$q_{di} = f\left( {\delta_{i} ,\sigma_{p} ,u_{i\max }^{{}} ,N_{sav} ,N_{bag} } \right)$$

where the parameters are equivalent to those in Eqs. (1) and (2). And dimensional analysis yields:

10 $$\frac{{q_{di} }}{{\sigma_{p} u_{i\max }^{{}} }} = f\left( {\delta_{i} ,\frac{{\sigma_{p} }}{{E_{i} }},N_{sav} ,N_{bag} } \right)$$

**Table 3:** Scale laws of the model tests.

Parameters	Dimension	Prototype/model
Gravity acceleration	*L*/*T*^2^	1
Froude number	–	1
Density	*M*/*L*^3^	1
Length	*L*	*n*
Velocity	*L*/*T*	*n*^0.5^
Distributed impact load *q*_*di*_	*M*/*T*^2^	*n*^2^
Impact stiffness* D*_*i*_	*M*/*LT*^2^	*n*

The left hand of Eq. (10) is $\frac{{D_{i} }}{{\sigma_{p} }}$. It is noteworthy that $\frac{{\sigma_{p} }}{{E_{i} }}$ is the pretension strain and remains constant in experimental tests, so *N*_*sav*_ and *N*_*bag*_ are focused in the data analysis. Since it is a preliminary deduction on the correlation between the two dimensionless parameters and the nondimensionalization of the impact stiffness, linear and power laws with no interceptions are chosen in data regression.

## Data Availability

The authors declare no competing interests.
